# Hepatitis B core‐related antigen levels predict recurrence‐free survival in patients with HBV‐associated early‐stage hepatocellular carcinoma: results from a Dutch long‐term follow‐up study

**DOI:** 10.1111/jvh.13394

**Published:** 2020-09-14

**Authors:** Boris J. B. Beudeker, Zwier M. A. Groothuismink, Robert A. de Man, Caroline D. M. Witjes, Annemiek A. van der Eijk, Andre Boonstra, Milan J. Sonneveld

**Affiliations:** ^1^ Department of Gastroenterology and Hepatology Erasmus MC University Medical Center Rotterdam The Netherlands; ^2^ Department of Surgery Erasmus MC University Medical Center Rotterdam The Netherlands; ^3^ Department of Viroscience Erasmus MC University Medical Center Rotterdam The Netherlands

**Keywords:** HBcrAg, hepatitis B, hepatocellular carcinoma, survival

## Abstract

Prognosis of hepatitis B (HBV)‐associated hepatocellular carcinoma (HCC) is poor due to high rates of HCC recurrence and progression of underlying liver disease. We studied whether serum hepatitis B core‐related antigen (HBcrAg) levels could predict HCC recurrence and outcome in HBV associated. Higher HBcrAg levels at HCC diagnosis were independently associated with reduced overall and recurrence‐free survival in patients with early, but not advanced, stage HCC.

## 1 INTRODUCTION

Chronic hepatitis B virus infection (HBV) affects more than 250 million people globally and is a leading cause of liver cirrhosis, liver failure and hepatocellular carcinoma (HCC). HCC is the fifth most common cause of cancer, and its long‐term outcome is poor even in patients undergoing potentially curative treatment, which is due to HCC recurrence and/or progression of the underlying liver disease.^1^ Currently, antiviral therapy with nucleo(s)tide analogues (NA) improves prognosis but does not cure HBV nor prevent HCC recurrence. The poor prognosis in HBV‐associated HCC may be partially attributed to persistence of intrahepatic replication and ongoing oncogenic processes that are not influenced by NA therapy mediated HBV DNA suppression. Hepatitis B core‐related antigen (HBcrAg) is an emerging serological biomarker that correlates with replicational activity of the intrahepatic cccDNA pool^2^ and has shown to predict HCC development in both NA treated and untreated patients.^3^, ^4^, ^5^, ^6^ Whether serum HBcrAg levels predict the recurrence‐free survival in patients with HBV‐associated HCC is unknown.

## 2 PATIENTS AND METHODS

### 2.1 Patient selection

Patients with chronic HBV and incident HCC visiting the outpatient clinic of the Erasmus Medical Center between 2000 and 2018 were identified. Patients were excluded in case of co‐infection with hepatitis C virus, hepatitis D virus, hepatitis E virus or human immunodeficiency virus, presence of auto‐immune liver disease, hemochromatosis, Wilson's disease or documented clinical history of alcohol abuse. Due to the retrospective nature of this study, written informed consent was not obtained. The ethical review board of the Erasmus Medical Center, Rotterdam, the Netherlands approved of the study as it was considered a low‐risk study using anonymized patient data, registered as MEC‐2017‐1140.

### 2.2 Data acquisition

Data were collected retrospectively from the electronic medical records. The diagnosis of HCC was either based on histopathology or imaging criteria in accordance with the international guidelines. We calculated follow‐up time from the date of HCC diagnosis until the date of death (ascertained through the national mortality database), or (for analyses on recurrence‐free survival) the date of disease recurrence based on noninvasive imaging‐based criteria or histology or the last date of the evaluation which was 20 November 2018.

Serum levels of HBcrAg (log U/mL) were measured using the Lumipulse G HBcrAg assay (Fujirebio Europe, Ghent, Belgium) on a LUMIPULSE G1200 analyser (Fujirebio Inc, Tokyo, Japan). The lower limit of detection was 2 log U/mL, with a validated lower limit of quantification of 3 log U/mL.^7^

### 2.3 Statistical analyses

SPSS version 25.0 (SPSS Inc, Chicago, IL, USA) was used for statistical analyses. Associations between variables were tested using Student's *t* test, chi‐square, Pearson correlation or their nonparametric equivalents when appropriate. We applied Kaplan‐Meier and Cox proportional hazard statistics to estimate the association between HBcrAg levels and other predictors with recurrence‐free survival and overall survival. Multivariate models were constructed using the enter method incorporating all significant factors from univariate analysis and clinically relevant factors, which comprised Child‐Pugh stage, age, alpha‐fetoprotein (AFP) levels, NA therapy, HBsAg levels and the exploratory variable HBcrAg. HBV DNA was not incorporated in the model due to collinearity with NA therapy. Analyses were performed in the overall population and in those with early‐stage HCC (BCLC stage 0 or A). All statistical tests were two‐sided and evaluated at the .05 level of significance.

## 3 RESULTS

### 3.1 Patient characteristics

A total of 119 patients with HBV‐associated HCC were included in the study, of whom 77% were males, mean age was 55 years, 68% were HBeAg‐negative, and 48% were on NA therapy at time of HCC diagnosis. Patients were Caucasian (51%), Asians (30%), or Africans (10%), and undefined in 9%. Patients had Barcelona Clinic Liver Cancer (BCLC) 0‐A/B/C/D stage in 53/36/10/20. All patients with an early HCC were treated with NA therapy after diagnosis. The median follow‐up time after HCC diagnosis was 22.0 months (IQR 4.9‐67.2). Seventy‐nine patients died during follow‐up. The 1‐, 3‐, 5‐ and 10‐year overall survival rates were 62%, 41%, 37% and 30%. Median HBcrAg levels at HCC diagnosis were 4.32 log U/mL (IQR 2.78‐5.82).

### 3.2 Factors associated with survival in the overall HBV‐associated HCC population

In univariate analysis, HBcrAg levels were not associated with survival (HR: 1.082, 95% CI: 0.948‐1.235, *P* = .243). Significant predictors of survival in the overall study population were BCLC stage (*P* < .0001), Child‐Pugh score (*P* < .0001), AFP levels (*P* < .0001), symptoms at time of diagnosis (*P* = .004) and NA therapy (*P* = .001). In multivariate analysis, only BCLC stage (*P* < .0001) and AFP levels (*P* = .003) were independently associated with survival.

**Figure 1:**
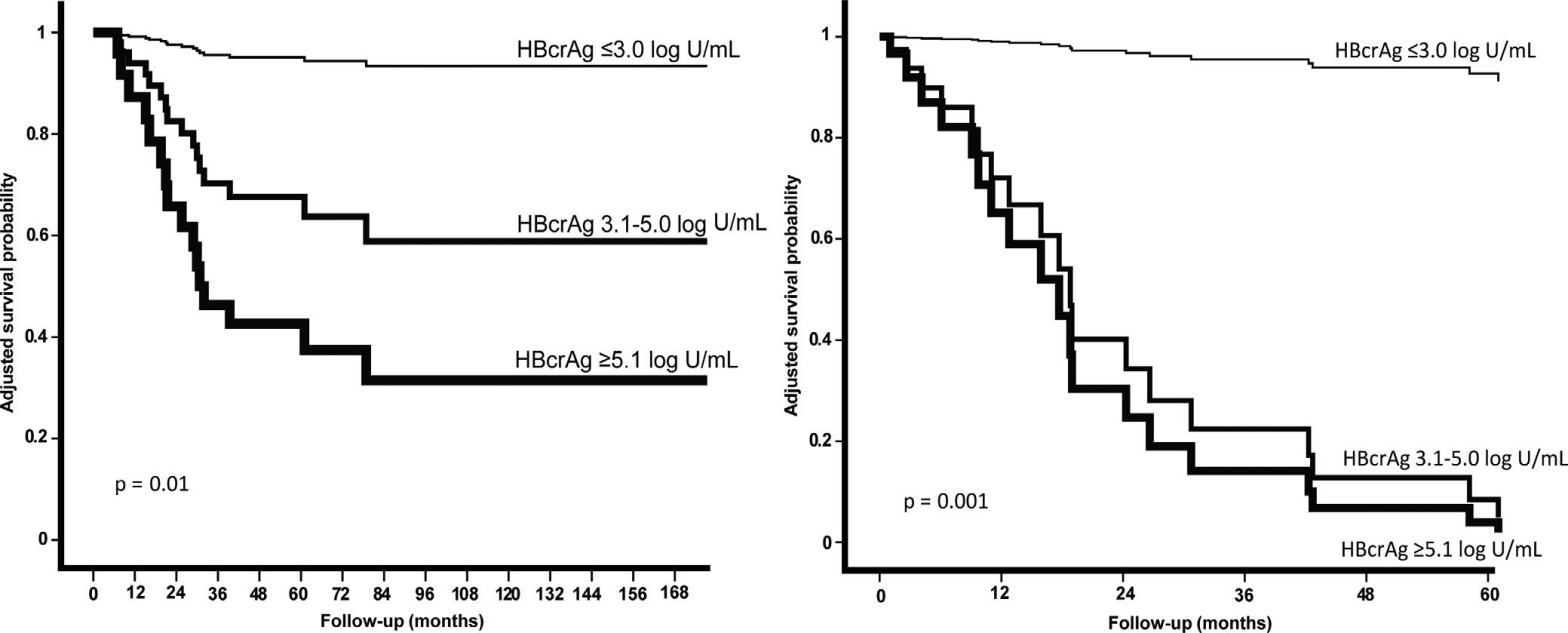
Adjusted overall survival (left) and recurrence‐free survival (right) according to HBcrAg level among patients with early‐stage HCC, stratified in terciles: (HBcrAg ≤ 3.0; HBcrAg 3.1‐5.0; HBcrAg ≥ 5.1)

Out of 119 patients, 53 patients were identified with early‐stage HCC (BCLC stage 0 or A) who received curative treatment: 27 (50%) surgical resection (wedge resection, segment resection or hemihepatectomy), 12 (23%) radio frequent ablation (RFA) and 14 (26%) liver transplantation. The 1‐, 3‐, 5‐ and 10‐year survival rates were 89%, 88%, 67% and 56%, respectively. Univariate analysis identified no predictors of survival, but in multivariate analysis, higher HBcrAg levels at the time of HCC diagnosis were independently associated with poorer overall survival (*P* = .01, Figure 1A), whereas age (*P* = .12), AFP levels (*P* = .58), Child‐Pugh score (*P* = .22), HBsAg levels (*P* = .11) and NA therapy (*P* = .38) were not.

Of the 39 (74%) early‐stage HCC patients receiving local therapy (RFA or resection), the median recurrence‐free survival was 33.6 months (range 12.5‐54.0). The 1‐, 3‐, 5‐ and 10‐year recurrence‐free survival rates were 72%, 48%, 39% and 35%, respectively. In univariate analysis, HBcrAg levels were not associated with the risk of recurrence (*P* = .91). In multivariate analysis HBcrAg levels (Figure 1B, *P* = .001), RFA (*P* = .001) and age (*P* = .030) were associated with reduced recurrence‐free survival, whereas AFP levels (*P* = .60), HBsAg levels (*P* = .22) and NA therapy (*P* = .16) were not.

## 4 DISCUSSION

Prognosis of HBV‐associated HCC remains poor due to limited options for curative treatment, high rates of post‐therapy recurrence and progression of underlying liver disease. In our cohort, of whom the majority were Caucasian, 5‐year survival was less than 37%, with BCLC stage being the main predictor for long‐term survival in the overall study population.

Recent studies have consistently shown a persistently elevated risk of HCC despite successful viral suppression in patients with chronic hepatitis B. This may be explained by ongoing intrahepatic viral replication that is not suppressed with NA therapy. This persistent replicational activity may be reflected by higher serum HBcrAg levels.^2^ Recent studies showing higher rates of HCC occurrence and reoccurrence in patients with higher HBcrAg levels during antiviral therapy provides further support for this hypothesis.^6^, ^8^ In the current study, HBcrAg levels at HCC diagnosis were not associated with HCC outcome in the overall population. Because the prognosis of patients with advanced‐stage HCC (BCLC stage B‐D) was dismal, with a median survival of less than 5 months, we also performed separate analyses in the subset of patients with early‐stage HCC. Among patients with early‐stage HCC, higher HBcrAg levels were independently associated with higher rates of HCC recurrence and higher risk of mortality. These findings in the subset of patients with early‐stage HCC are important, since they provide further support for the concept that complete viral eradication is necessary to improve outcomes, and it confirms the association between HBcrAg and HCC risk. Also, HBcrAg levels might potentially help identify patients with high rates of disease recurrence who may benefit more from liver transplant than local therapy because of their high recurrence risk after local therapy. Our findings also suggest that among those with advanced‐stage HCC, virological factors are probably less important and prognosis is dependent on the (often already advanced) stage of liver disease.

Our study is quite unique since it enrolled mainly non‐Asian patients with HBV‐associated HCC with a very long follow‐up duration. However, there are some limitations. The most important is the relatively limited number of patients, especially after stratification by BCLC stage. However, our findings are robust, which is most clearly reflected in the comparable outcomes regarding both HCC recurrence and overall survival. Nevertheless, this was a single‐centre study and further external validation of our findings is therefore required. Given that we mainly enrolled Caucasian patients, confirmation is also required in patients with other ethnicities.

In conclusion, serum HBcrAg levels are independently associated with overall and recurrence‐free survival in patients with early, but not advanced, stages of, HBV‐associated HCC.

## CONFLICT OF INTEREST

MJS received research support and consultancy fees from Fujirebio. AB received research support from Fujirebio. The other authors report no disclosures.
